# Loading Patterns of the Posterior Cruciate Ligament in the Healthy Knee: A Systematic Review

**DOI:** 10.1371/journal.pone.0167106

**Published:** 2016-11-23

**Authors:** S. H. Hosseini Nasab, Renate List, Katja Oberhofer, Sandro F. Fucentese, Jess G. Snedeker, William R. Taylor

**Affiliations:** 1 Institute for Biomechanics, ETH Zürich, Zürich, Switzerland; 2 University Hospital Balgrist, Zürich, Switzerland; Queen Mary University of London, UNITED KINGDOM

## Abstract

**Background:**

The posterior cruciate ligament (PCL) is the strongest ligament of the knee, serving as one of the major passive stabilizers of the tibio-femoral joint. However, despite a number of experimental and modelling approaches to understand the kinematics and kinetics of the ligament, the normal loading conditions of the PCL and its functional bundles are still controversially discussed.

**Objectives:**

This study aimed to generate science-based evidence for understanding the functional loading of the PCL, including the anterolateral and posteromedial bundles, in the healthy knee joint through systematic review and statistical analysis of the literature.

**Data sources:**

MEDLINE, EMBASE and CENTRAL

**Eligibility criteria for selecting studies:**

Databases were searched for articles containing any numerical strain or force data on the healthy PCL and its functional bundles. Studied activities were as follows: passive flexion, flexion under 100N and 134N posterior tibial load, walking, stair ascent and descent, body-weight squatting and forward lunge.

**Method:**

Statistical analysis was performed on the reported load data, which was weighted according to the number of knees tested to extract average strain and force trends of the PCL and identify deviations from the norms.

**Results:**

From the 3577 articles retrieved by the initial electronic search, only 66 met all inclusion criteria. The results obtained by aggregating data reported in the eligible studies indicate that the loading patterns of the PCL vary with activity type, knee flexion angle, but importantly also the technique used for assessment. Moreover, different fibres of the PCL exhibit different strain patterns during knee flexion, with higher strain magnitudes reported in the anterolateral bundle. While during passive flexion the posteromedial bundle is either lax or very slightly elongated, it experiences higher strain levels during forward lunge and has a synergetic relationship with the anterolateral bundle. The strain patterns obtained for virtual fibres that connect the origin and insertion of the bundles in a straight line show similar trends to those of the real bundles but with different magnitudes.

**Conclusion:**

This review represents what is now the best available understanding of the biomechanics of the PCL, and may help to improve programs for injury prevention, diagnosis methods as well as reconstruction and rehabilitation techniques.

## Introduction

The posterior cruciate ligament (PCL) is one of the major passive stabilizers of the knee joint, serving as the primary restraint to excessive posterior tibial translation, but is also thought to act as a secondary restraint to tibial rotation [1, 2]. It is likely that injuries to the PCL interfere with its normal function and consequently lead to joint instability [3], as well as to possible subsequent osteoarthritis [4]. In order to better understand these processes, as well as improve diagnosis, reconstruction and rehabilitation techniques, a number of studies have investigated healthy PCL loading and injury biomechanics [5–22]. Here, the key goal is to quantify the physiological loading patterns of the healthy PCL during sport and activities of daily living. However, despite a number of *in vitro* [23–29], *in vivo* [30–33], and modelling [34–38] approaches to provide science based evidence, the normal loading conditions of the PCL are still controversially discussed [2, 5, 39].

Although the PCL is the strongest ligament of the knee joint [40, 41], some scientists have dismissed it as functionally superfluous [32, 42, 43]. These ideas originated mainly from studies reporting either no or very small forces in the PCL during activities of daily living [32, 37, 44] and partially from those stating that an isolated PCL tear may not interfere with patient movement [45, 46]. On the other hand, a number of studies have reported PCL loads very close to or even higher than the failure limit of the ligament during activities such as passive knee flexion, body weight squats and forward lunge [14, 47–49]. These contrasting reports most likely result from the difficulties in accessing the kinematics and kinetics of the ligament *in vivo*. Nevertheless, this controversy indicates a clear requirement for improved understanding of the PCL and its functionality, which is closely related to the biomechanical function and loading behaviour of its anterolateral (AL) and posteromedial (PM) bundles. Unfortunately, a comprehensive investigation regarding the contributions of each bundle in providing stability to the knee is lacking. Traditionally, the two bundles were mainly believed to have reciprocal functions during flexion and extension of the knee [6, 50]. This argument has been supported by a number of studies reporting lengthening of the AL bundle and shortening of the PM bundle during knee flexion [10, 27, 29]. However, more recently, a co-dominant functional relationship between the two bundles has been suggested based on simultaneous elongation observed in both PCL bundles during a forward lunge [19, 25, 51].

The reasons for the lack of knowledge regarding loading of the PCL are manifold. Most importantly, PCL injuries have been historically underdiagnosed due to the high possibility of asymptomatic damage [52], and therefore the necessity to investigate PCL biomechanics has been generally underestimated. Over the last two decades, it has become apparent that PCL injuries are more prevalent than previously believed, with its involvement in nearly 3% of all knee injuries [53], and 38% of acute knee injuries [54]. Considering these findings, together with an increasing number of vehicular traumas as the main cause of PCL rupture, scientists and clinicians have become more interested in research regarding the functional loading of the PCL within the knee joint [55].

A number of technical challenges have also limited the accurate *in vivo* assessment of PCL function during dynamic movement. Compared to other ligaments of the knee, investigation into the function and loading patterns of the PCL is difficult due to its relatively inaccessible anatomical location within the knee joint. Furthermore, the PCL possesses a complex fibrous anatomy and undergoes differing contact conditions with neighbouring bone and soft tissue structures, all leading to kinematics that are difficult to assess [14, 22, 56]. As a consequence, a number of both direct (sensor-based) and indirect (predominantly image-based) approaches have been used to examine the behaviour of the PCL using *in vivo*, *in vitro* and modelling investigations. The most common *in vivo* approach to determine strain has been to examine the relative movement of the bony attachment sites of the PCL [14, 30, 32, 33, 57]. Here, the length of virtual bundles are determined as the absolute distance between the centroids of the origin and insertion sites, while virtual bundle strain (VBS) has been defined as the elongation from the reference length, which is generally selected as the length of the ligament at full extension of the knee, relative to their length measured at different flexion angles. Here, CT and MR imaging, and even dual-plane fluoroscopy, are generally used to track the attachment sites of the bundles.

Given the hurdles associated with *in vivo* studies, current knowledge of PCL loading has mainly been based on *in vitro* cadaveric investigations [23, 25, 26, 28, 29, 49, 58–61]. Here, strain and force sensors combined with either mechanical jigs or robotic manipulators have proven to be successful approaches for investigating the PCL. However, due to the complexities of applying physiological loading conditions to *in vitro* set-ups, the measurement of force or strain in the PCL has been generally limited to passive flexion [13, 20, 26, 62, 63], flexion under externally applied loads [13, 26, 62, 64, 65], or with simplified muscle forces only [26, 66–68]. Compared to the assessment of strain, force is considered to provide a more realistic measure of the ligament load [69]; however, studies with implanted force sensors suffer from the imposed size and stiffness of the sensors, which compromise the natural kinematics and loading within the ligament. To avoid any interference caused by implantable sensors, an indirect method using robotic imposed kinematics has also been introduced for estimating total ligament force based on the principle of superposition [15, 56, 59]. Although this approach allows a contact-less measurement of the effect of the ligament force, the in situ forces obtained do not necessarily represent the exact ligament forces [70]. Strain sensors have less influence on the normal ligament behaviour due to their small size and low stiffness and therefore have been frequently used in cadaveric investigations of PCL loading [49, 58, 71–73]. The sensor output or real bundle strain (RBS) represents the relative elongation of the real curvilinear bundle path of the ligament. However, due to the locality of the sensor measurement site, together with the invasive nature of sensor implantation, researchers have recently turned to indirect approaches for strain measurement. When used in cadaveric studies, these indirect methods have the advantage that they are not limited to image-based assessment. To obtain more accurate results, 3D coordinate measuring machines [19, 74] and surgical navigation systems [23, 25] have also been introduced into cadaveric investigations of ligament strain.

In addition to experimental studies, *in silico* musculoskeletal and finite element modelling studies [34–37, 75] have also reported loading behaviour of the PCL. Musculoskeletal models have been generally used to estimate the PCL force patterns during dynamic physiological activities such as walking [76–78] and squatting [47, 48, 79]. In contrast, finite element models of the knee have been mostly subjected to either static or quasi-static loading conditions [36, 80, 81]. Despite their ability to strongly complement *in vivo* and *in vitro* approaches, modelling investigations have suffered from oversimplifications of the structural geometries, material properties and loading conditions. For example, in the vast majority of models reported in the literature, ligaments have been represented using one-dimensional elements which therefore preclude the prediction of non-uniform strain distributions within the three-dimensional structure of the ligament [82].

Improvements in modelling and experimental techniques have provided greater insights into the functional loading of the PCL in the healthy knee joint. However, differing results from *in vivo*, *in vitro* and *in silico* studies remain controversially discussed and a clear unbiased consensus on the biomechanics of the functional bundles for the healthy knee is still lacking. In particular, the following three questions need to be comprehensively answered: (1) Do the AL and PM bundles have different loading patterns during loaded and unloaded flexion of the knee? (2) Is the loading behaviour of the functional bundles of the PCL activity-dependent? (3) Are the observed loading patterns dependent upon the investigation techniques used to assess them? This study therefore aims to generate evidence-based understanding of the functional loading of the PCL in the healthy knee joint, including its AL and PM bundles, through systematic review and statistical analysis of the current literature.

## Methods

### Literature Search and Study Selection

For the systematic review, the databases PubMed, Embase, and Cochrane Central Register of Controlled Trials (CENTRAL) were all searched from their inception up to January 2016 to recruit studies that reported load data (strain or force) for the PCL. Different combinations of the terms “knee”, ‘‘ligament”, ‘‘load”, ‘‘force”, “tension“, “length”, ‘‘strain”, “elongation” and ‘‘lengthening” were used. To facilitate collection of the systematic review manuscripts as well as identification of duplicate reports, all search hits were imported into the Eppireviewer software (version 4.5.0.1) [83]. Titles and abstracts of all search hits were screened for eligibility based on the inclusion/exclusion criteria as follows:

### Inclusion criteria

- Subject characteristics
  Human living subjects or cadaveric specimens with healthy ligaments inside healthy knees
- Bundle definition
  Common anatomical definition was used, which consisted of real or virtual anterolateral (AL), posteromedial (PM) bundles [2], and the mid-PCL fibre as a representative of the whole PCL
- Activities
  Physiological activities including passive flexion, body-weight squat, forward lunge, walking, stair ascent, stair descent
  Clinical tests including passive flexion with 100 N or 134 N posterior tibial load (PTL)
- Results
  Numeric strain or force data
  Length or elongation of the bundles when the reference length was specified
- Report
  Journal articles and conference proceedings with full texts written in the English language

### Exclusion criteria

- Subject characteristics

Animal subjects or specimens, deficient or reconstructed ligaments, pathologic knees

- Load measurement techniques

Techniques that provided no quantitative data of ligament strain or force (e.g. arthroscopic probes to assess the slack/tense status of the ligament)

- Bundle definition

Virtual bundles that did not match anatomical structure

- Results

Data collected using non-calibrated sensor outputs

Strain data where the reference length was not reported

Duplicate results (including multiple studies that report on the same cohorts)

- Report

Articles without full texts, non-English reports, and review papers

After initial screening to reduce the pool according to clearly met criteria for exclusion, the final decision to include or exclude a specific study was made after carefully reading the full text article. The reference lists of all included full text articles were double-checked to identify additional reports that were possibly missed in the initial systematic search (e.g. in cases where reporting ligament strains and forces was not the primary focus of the manuscript).

### Analysis of the Literature

An analysis was conducted to obtain the average load trends of the PCL and its two functional bundles during passive and active knee flexion, as well as to compare the reported strain and force results between direct and indirect methods. Outcome measures used to assess the loading trends of the PCL predominantly included measures of strain and force. It should be noted that due to varying definitions of ligament physiological length, which relied upon lax versus taught, knee flexion angle and insertion site definitions, the term “strain” alone was not sufficient to report and compare the results from the different methodologies. Therefore, we introduce the term real bundle strain (RBS) to address the strain within the PCL that included the real curvilinear path of the ligament. RBS can be measured either using implanted strain sensors or by calculating the relative elongation of a curvilinear fibre that follows the centroid-axis of the bundle based on medical images. Similarly, the term virtual bundle strain (VBS) was used to describe the relative elongation of a virtual straight-line fibre connecting the centroids of the two bony attachment sites of the entire ligament or an individual bundle. VBS can be determined by tracking and assessing the relative change in distance between the ligament origin and insertion sites (using e.g. medical images or data from coordinate measurement systems). As with strain data, the reported force data were also obtained using different techniques. In this review, the term “force” was used to describe ligament tensile forces, as measured by force sensors or calculated by means of computational modelling, while “*in situ* force” was used to represent forces obtained based on the principle of superposition.

Real and virtual bundle strains, force and *in situ* force data of the PCL were thus extracted from the included articles. If numeric data were not explicitly reported within the article, graphs were carefully digitized for data extraction. It should be noted that while the strain and force data during walking and stair activities were included in the systematic review for qualitative comparison of the literature, the data were excluded from the statistical analysis because of the inconsistent formats used for reporting results. In order to be able to compare the data from the different studies, the reported measures of length were converted into measures of strain by dividing the length changes by the given reference length (bundle length at full extension). To extract the strain patterns of the PCL bundles during passive flexion, a weighted-regression approach was used based on the number of knees tested in the various studies. The average load trends of the PCL bundles were then extracted for the different activities using the weighted mean and standard error of the mean (SEM; presented in parenthesis after the mean value) of the extracted force and strain data at the most frequently reported angles of 0°, 30°, 60°, 90° and 120° of knee flexion.

To reduce the risk of bias, all studies with at least one author in common were assigned to a specific research group. The articles that belonged to specific research groups were then assessed carefully to ensure that subjects or cohorts were not reported on more than one occasion. Those articles with no evidence of duplicate data were removed from the specific research group and were analysed using the routine procedure as outlined above. The data from articles with duplicate results were averaged and only the mean trend of each group was included in the statistical analysis.

## Results

### Study Selection

**Fig 1:**
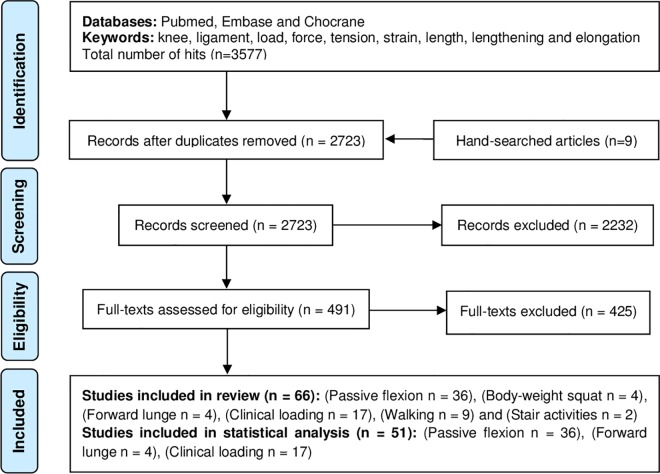
The 2009 PRISMA flowchart for the systematic review.

A total of 3577 articles were found through the initial electronic search. Search results were imported into the Eppireviewer software, where 854 items were identified as duplicates. An additional 2232 studies were excluded after initial screening. The full-texts of the remaining 491 studies were retrieved for further evaluation. Here, evaluation was conducted to ensure inclusion of articles that contained PCL load data even if the main focus of a study was on another ligament. A comprehensive full-text screening revealed that 66 articles contained strain or force data regarding the PCL (including 9 eligible manuscripts that were found through double-checking the reference lists of the included full-text articles), and thus fulfilled all inclusion criteria (Fig 1; data classified according to the activity types in Tables 1–4).

**Table 1:** Summary of the characteristics of the systematic review studies that address passive knee flexion.

Ref.	Lead author (year)	Study method	Testing apparatus	Measurement technique	Subjects characteristics	Studied bundles	Outcome
[84]	Amiri (2007)	Modelling		3D quasi-static modelling		AL, PM and Mid-PCL	Virtual bundle strain/tension
[82]	Amiri (2011)	*In vitro* cadaveric	6 DOF Axford rig	Indirect strain measurement (registering 3D geometry on to joint kinematics)	3 knees with intact soft tissues	AL, PM and Mid-PCL	Virtual bundle strain
[49]	Arms (1984)	*In vitro* cadaveric		Direct strain measurement (using HEST)	3 knees (details not reported)	AL and PM	Real bundle strain
[23]	Belvedere (2012)	*In vitro* cadaveric	Manual rig	Indirect strain measurement (registering 3D geometry on to joint kinematics)	10 full legs with intact soft tissues (31<age<92)	AL and PM	Virtual bundle strain
[75]	Beynnon (1996)	Modelling		2D quasi-static modelling		AL and PM	Virtual bundle strain/tension
[24]	Blankevoort (1991)	*In vitro* cadaveric	Multi-DOF manual rig	Indirect strain measurement (tracking of attachment sites using RSA)	4 knees with intact soft tissues (43<age<74)	AL and PM	Virtual bundle strain
[35]	Chittajallu (1996)	Modelling		2D dynamic modelling		Mid-PCL	Virtual bundle strain
[25]	Cross (2013)	*In vitro* cadaveric	Details not reported	Indirect strain measurement (registering 3D geometry on to joint kinematics)	7 knees (25<age<93)	AL and PM	Virtual bundle strain
[34]	Crowninshield (1976)	Modelling		3D kinematic modelling		AL and PM	Virtual bundle strain
[73]	Dorlot (1983)	*In vitro* cadaveric	Details not reported	Indirect strain measurement (measuring distance between attachment sites using displacement transducers)	15 knees with resected soft tissues (except ligaments)	AL and PM	Virtual bundle strain
[26]	Dürselen (1995)	*In vitro* cadaveric	Multi-DOF mechanical rig	Direct strain measurement (using Ω-shaped strain sensors)	9 knees with resected skin and muscles (22<age<55)	PM	Real bundle strain
[51]	Garbelotti (2007)	*In vitro* cadaveric	1 DOF Manual rig	Indirect strain measurement (tracking of attachment sites using RSA)	18 knees with intact capsule and ligaments	AL and Mid-PCL	Virtual bundle strain
[9]	Höher (1999)	*In vitro* cadaveric	Robot with UFS	Indirect force measurement (using principal of superposition)	9 knees with intact soft tissues (52<age<86)	Mid-PCL	*In situ* force
[27]	Hsieh (1997)	*In vitro* cadaveric	Multi DOF manual rig	Indirect strain measurement (registering 3D geometry on to joint kinematics)	15 knees with intact soft tissues and resected skins (28<age<76)	AL, PM and other	Virtual bundle strain
[60]	Inderster (1995)	*In vitro* cadaveric		Indirect strain measurement (measuring distance between attachment sites using an isometer)	10 knees with intact capsule and ligaments	AL, PM and other	Virtual bundle strain
[85]	Jeong (2010)	*In vivo*		Indirect strain measurement (tracking of attachment sites using 3D CT-imaging)	10 living subjects (21<age<39)	AL and PM	Virtual bundle strain
[31]	King (2012)	*In vivo*		Indirect strain measurement (tracking of attachment sites using open-bore MRI)	7 living subjects (18<age<65)	AL and PM	Real bundle strain
[63]	Lew (1982)	*In vitro* cadaveric	Investigator hand	Direct strain measurement (using buckle force transducers)	3 knees with intact capsule and ligaments	PM	Tension
[13]	Markolf (1997)	*In vitro* cadaveric	Multi DOF manual rig	Direct force measurement (using load cells attached to the femoral insertion of the PCL)	12 knees (details not reported)	Mid-PCL	Tension
[86]	Markolf (2004)	*In vitro* cadaveric	Multi-DOF manual rig	Direct force measurement(using load cells attached to the femoral insertion of the PCL)	13 knees (29<age<67)	Mid-PCL	Tension
[87]	Markolf (2006)	*In vitro* cadaveric	Multi-DOF manual rig	Direct force measurement (using load cells attached to the femoral insertion of the PCL)	16 knees (Mean age = 38.4)	Mid-PCL	Tension
[88]	Markolf (2006)	*In vitro* cadaveric	Multi-DOF manual rig	Direct force measurement (using load cells attached to the femoral insertion of the PCL)	13 knees (22<age<65)	Mid-PCL	Tension
[12]	Markolf (2010)	*In vitro* cadaveric	Multi-DOF manual rig	Direct force measurement (using load cells attached to the femoral insertion of the PCL)	10 knees (21<age<45)	Mid-PCL	Tension
[20]	Wascher (1993)	*In vitro* cadaveric	Multi DOF manual rig	Direct force measurement (using load cells attached to the femoral insertion of the PCL)	18 knees (48<age<74) with surrounding soft tissues	Mid-PCL	Tension
[62]	Miyasaka (2002)	In-vitro cadaveric	*In vitro* cadaveric	Direct force measurement (using load cells attached to the femoral insertion of the PCL)	6 knees with intact capsule and ligaments (36<age<86)	Mid-PCL	Tension
[36]	Moglo (2005)	Modelling		Quasi-static Finite Element Analysis		Mid-PCL	Tension
[32]	Nakagawa (2004)	*In vivo*		Indirect strain measurement (tracking of attachment sites using MRI)	13 living subjects (mean age = 26)	Mid-PCL	Virtual bundle strain
		*In vitro* cadaveric		Indirect strain measurement (tracking of attachment sites using MRI)	6 knees with intact capsule and ligaments (mean age = 43)	Mid-PCL	Virtual bundle strain
[89]	Oakes (2003)	*In vitro* cadaveric	Multi-DOF manual rig	Direct force measurement (using load cells attached to the isolated femoral insertion)	9 knees (43<age<69)	Mid-PCL	Tension
[61]	Trent (1976)	*In vitro* cadaveric	Manual rig	Indirect strain measurement (registering 3D geometry on to joint kinematics)	10 knees (29<age<55)with intact soft tissues and resected skins	AL, PM and Mid-PCL	Virtual bundle strain
[29]	Van Dijk (1979)	*In vitro* cadaveric		Indirect strain measurement (tracking of attachment sites using RSA)	2 knees (60<age<79) with intact capsule and ligaments	AL and PM	Virtual bundle strain
[90]	Wang (1973)	*In vitro* cadaveric		Indirect strain measurement (tracking of attachment sites using RSA)	12 knees with intact capsule and ligaments	Mid-PCL	Virtual bundle strain
[91]	Wang (2002)	*In vitro* cadaveric	Multi-DOF manual rig	Direct force measurement (using load cells attached to the isolated tibial insertion)	12 knees with intact ligaments	Mid-PCL	Tension
[19]	Wang (2014)	*In vitro* cadaveric	Robot with UFS	Indirect force measurement (using principal of superposition)	10 knees (50<age<65) with intact soft tissues	AL, PM and Mid- PCL	Virtual bundle strain
[92]	Wismans (1980)	Modelling		3D quasi-static modelling		Mid-PCL	Virtual bundle strain
[22]	Zaffagnini (2004)	*In vitro* cadaveric	Manual rig	Indirect strain measurement (registering 3D geometry on to joint kinematics)	8 knees with partially resected soft tissues	AL and PM	Virtual bundle strain
[38]	Zavatsky (1992)	Modelling		2D kinematic modelling		AL and PM	Virtual bundle strain

Abbreviations: AL–Anterolateral; DOF–Degrees of Freedom; HEST–Hall Effect Strain Transducer; MRI–Magnetic Resonance Imaging; PCL–Posterior Cruciate Ligament; PM–Posteromedial; RSA–Roentgen Stereophotogrammetric Analysis; UFS–Universal Force Sensor

**Table 2:** Summary of the characteristics of the systematic review studies that address passive flexion with 100 or 134 N posterior tibial load.

Ref.	Lead author (year)	Study method	Testing apparatus	Measurement technique	Subjects characteristics	Studied bundle	Posterior tibial load	Outcome
[8]	Harner (2000)	*In vitro* cadaveric	Robot with UFS	Indirect force measurement (using principal of superposition)	10 knees (39<age<73) with intact soft tissues	PCL	134 N	*In situ* force
[93]	Harner (2000)	*In vitro* cadaveric	Robot with UFS	Indirect force measurement (using principal of superposition)	10 knees (36<age<65) with intact soft tissues	PCL	134 N	*In situ* force
[94]	Harner (2000)	*In vitro* cadaveric	Robot with UFS	Indirect force measurement (using principal of superposition)	10 knees (44<age<71) with intact soft tissues	PCL	134 N	*In situ* force
[95]	Margheritini (2005)	*In vitro* cadaveric	Robot with UFS	Indirect force measurement (using principal of superposition)	10 knees (34<age<80) with intact soft tissues	PCL	134 N	*In situ* force
[16]	Sekiya (2005)	*In vitro* cadaveric	Robot with UFS	Indirect force measurement (using principal of superposition)	10 knees (38<age<71) with intact soft tissues	PCL	134 N	*In situ* force
[18]	Vogrin (2000)	*In vitro* cadaveric	Robot with UFS	Indirect force measurement (using principal of superposition)	10 knees (52<age<86) with intact soft tissues	PCL	134 N	*In situ* force
[12]	Markolf (2010)	*In vitro* cadaveric	Multi-DOF manual jig	Direct force measurement (using load cells attached to the femoral insertion of the PCL)	10 knees (21<age<45)	PCL	100 N	Tension
[96]	Markolf (2007)	*In vitro* cadaveric	Multi DOF manual jig	Direct force measurement (using load cells attached to the femoral insertion of the PCL)	12 knees (17<age<65)	PCL	100 N	Tension
[87]	Markolf (2006)	*In vitro* cadaveric	Multi DOF manual jig	Direct force measurement (using load cells attached to the femoral insertion of the PCL)	16 knees (mean age = 38.4)	PCL	100 N	Tension
[28]	Markolf (2006)	*In vitro* cadaveric	Multi-DOF manual Jig	Direct force measurement (using load cells attached to the femoral insertion of the PCL)	13 knees (22<age<65)	PCL	100 N	Tension
[86]	Markolf (2004)	*In vitro* cadaveric	Multi-DOF manual Jig	Direct force measurement (using load cells attached to the femoral insertion of the PCL)	13 knees (29<age<67)	PCL	100 N	Tension
[13]	Markolf (1997)	*In vitro* cadaveric	Multi DOF manual jig	Direct force measurement (using load cells attached to the femoral insertion of the PCL)	12 knees	PCL	100 N	Tension
[97]	Markolf (1996)	*In vitro* cadaveric	Multi DOF manual jig	Direct force measurement (using load cells attached to the femoral insertion of the PCL)	15 knees (63<age<84)	PCL	100 N	Tension
[89]	Oakes (2003)	*In vitro* cadaveric	Multi-DOF manual Jig	Direct force measurement (using load cells attached to the femoral insertion of the PCL)	9 knees (43<age<69)	PCL	100 N	Tension
[15]	Petersen (2006)	*In vitro* cadaveric	Robot with UFS	Indirect force measurement (using principal of superposition)	10 knees (54<age<78) with intact soft tissues	PCL	134 N	*In situ* force
[98]	Lenschow (2006)	*In vitro* cadaveric	Robot with UFS	Indirect force measurement (using principal of superposition)	10 knees (49<age<78) with resected soft tissues	PCL	134 N	*In situ* force
[17]	Vahey (1991)	*In vitro* cadaveric	Milling machine with a load cell	Indirect force measurement (using principal of superposition)	6 knees (38<age<84) with intact soft tissues	PCL	100 N	*In situ* force

Abbreviations: DOF–Degrees of Freedom; PCL–Posterior Cruciate Ligament; UFS–Universal Force Sensor

**Table 3:** Summary of the characteristics of the reviewed studies that address forward lunge or body-weight squat.

Ref.	Lead author (year)	Study method	Measurement technique	Subjects characteristics	Studied bundles	Activity	Outcome
[30]	Defrate (2004)	*In vivo*	Indirect strain measurement (registering 3D geometry on to joint kinematics)	5 living subjects (mean age = 25)	Mid-PCL	Forward lunge	Virtual bundle strain
[57]	Li (2004)	*In vivo*	Indirect strain measurement (registering 3D geometry on to joint kinematics)	5 living subjects (mean age = 25)	AL and PM	Forward lunge	Virtual bundle strain
[14]	Papannagari (2007)	*In vivo*	Indirect strain measurement (registering 3D geometry on to joint kinematics)	7 living subjects (22<age<44)	AL and PM	Forward lunge	Virtual bundle strain
[33]	Yue (2012)	*In vivo*	Indirect strain measurement (registering 3D geometry on to joint kinematics)	22 living subjects (51<age<73)	AL and PM	Forward lunge	Virtual bundle strain
[32]	Nakagawa (2004)	*In vivo*	Indirect strain measurement (tracking of attachment sites using MRI)	13 living subjects (mean age = 26) living subjects	Mid-PCL	Body weight squat	Virtual bundle strain
[37]	Shelburne (2011)	Modelling	3D musculoskeletal modelling		Mid-PCL	Body weight squat	Tension
[99]	Shelburne (1998)	Modelling	2D musculoskeletal modelling		Mid-PCL	Body weight squat	Tension
[47]	Toutoungi (2000)	Modelling	2D musculoskeletal modelling		Mid-PCL	Body weight squat	Tension

Abbreviations: AL–Anterolateral; PCL–Posterior Cruciate Ligament; PM–Posteromedial; MRI–Magnetic Resonance Imaging

**Table 4:** Summary of the characteristics of the reviewed studies that address walking, stair ascent or stair descent.

Ref.	Lead author (year)	Study method	Measurement technique	Subjects characteristics	Studied bundles	Activity	Maximum ligament load
[100]	Belbasis (2015)	modelling	2D forward dynamic modelling		PCL	Walking	253 N maximum ligament force at 95% stance phase
[101]	Collins (1991)	modelling	2D musculoskeletal modelling		PCL	Walking	0.3 BW maximum ligament force at 85% gait cycle
[77]	Collins (1995)	modelling	2D musculoskeletal modelling		PCL	Walking	0.2–0.6 BW maximum ligament force
[58]	Emodi (1999)	*In vitro* cadaveric	Direct strain measurement (using DVRT)	4 knees (40<age<79) with intact capsule and ligaments	AL	An active flexion between 15° to 105° (a simulated stair descent)	8.7±1.7% maximum real bundle strain at 105° flexion
[102]	Harrington (1976)	modelling	3D musculoskeletal modelling		PCL	Walking	375 N maximum ligament force at late stance
[103]	Hu (2013)	modelling	3D musculoskeletal modelling		PCL	Walking	0.8–1 BW maximum ligament force at late stance
[71]	Mahoney (1994)	*In vitro* cadaveric	Direct strain measurement (using HEST)	8 knees with intact capsule and ligaments	AL	An active flexion between 0° to 120° (a simulated stair ascent)	2.6 ±0.1% maximum real bundle strain at 100°-110° flexion
						An active flexion between 0° to 110° (a simulated stair descent)	2.5 ±0.1% maximum real bundle strain at 100°-110° flexion
[78]	Morrison (1970)	modelling	3D musculoskeletal modelling		PCL	Walking	330 N at early stance
[104]	Shelburne (2004)	modelling	3D musculoskeletal modelling		PCL	Walking	27 N at 85% gait cycle
[37]	Shelburne (2011)	modelling	3D musculoskeletal modelling		PCL	Walking	PCL was unloaded during walking
[44]	Yang (2010)	modelling	Finite Element Analysis		PCL	Walking	3 N maximum ligament force at 60% stance phase

Abbreviations: AL–Anterolateral; BW–Body Weight; DVRT–Differential Variable Reluctance Transducer; HEST–Hall Effect Strain Transducer; PCL–Posterior Cruciate Ligament

### Strain Patterns of the PCL during Passive Flexion

Twenty-four studies that contained PCL strain data during passive flexion met all inclusion criteria; from those, only three studies assessed the RBS within the PCL, whereas in the remaining experimental studies a variety of indirect methods was used for measuring the VBS. Most studies (16 articles) presented *in vitro* cadaveric approaches, while modelling (6 articles) and *in vivo* (3 articles) methods were less frequently utilised.

#### In vivo results

Only three studies reported on strain of the PCL during passive flexion *in vivo*. Jeong and co-workers [85] calculated the distances between the attachment sites of the AL and PM bundles using CT images of ten living subjects. At 90° of flexion, the AL and PM bundles were elongated 33% and 9% respectively compared to their reference lengths at full extension of the knee. From 90° to 135° of flexion, there was no considerable further lengthening in the AL bundle, but the PM bundle continued its elongation. In another study, based on MR images of twenty subjects in the sagittal plane, Nakagawa et al. [32] reported lengthening of the virtual bundles that represented the mid-PCL. With the reference length taken at full extension, the VBS trend was upward, with a calculated strain of 29% at 90°. Further flexion beyond 90° caused a reduction of the PCL strain to a value of 24% at 120°. These findings are in general agreement with those reported by King and co-workers [31] who used an open-bore MRI scanner to measure lengthening of the virtual mid-PCL bundle in seven subjects. In addition to the virtual mid-PCL bundle, the elongation of the curvilinear fibres on the anterior and posterior surfaces of the PCL was calculated. Their results indicated continuous elongation of the anterior fibre up to 22% at 120°, while the posterior fibre lengthened up to 40° but became shorter thereafter.

#### In vitro results

Two studies reported RBS data for the PCL during passive flexion of the knee. Arms and co-workers [49] attached Hall Effect Strain Transducers (HESTs) to the anterior and posterior fibres of the PCL in cadaveric specimens. They found a rapid elongation of the anterior bundle after the first 10° of flexion, corresponding to 19% maximum strain at 120°. The posterior bundle was positively strained only after 45° of flexion and gradually elongated thereafter. In another sensor-based study, Dürselen and co-workers [26] attached Ω-shaped strain transducers to the PM bundle of the PCL in nine cadaveric specimens. Since their study considered the zero-strain position to be at 60° flexion, this point was referenced to full extension in order to allow an objective comparison of their results. Given this transformation, the PM bundle was shorter than its reference length up to 30° flexion, whereupon the strain gently increased up to 1% at 110°, with the minimum strain observed at 15° flexion.

Other *in vitro* studies have used indirect techniques for measuring the PCL strain in cadaveric knees. Dorlot and co-workers [73] as well as Inderster and co-workers [60] measured the distance between origin and insertion of the anterior and posterior fibres of the PCL using threads passed through the centres of the attachment sites with one end secured to the tibia and the other connected to a displacement transducer. They found a gradual elongation of the anterior bundle throughout the full range of knee flexion but the reported strain patterns of the posterior bundle were not consistent in these studies. While in the first study [73] the posterior bundle was found slack throughout the first 50° flexion, the second study [60] reported a continuous elongation of the bundle with increasing knee flexion. Using Rontgen Stereo-photogrammetric Analysis (RSA) to measure the VBS of the PCL in cadaveric knees, Garbelotti and co-workers [51], van Dijk and co-workers [29], as well as Blankevoort and co-workers [24] reported lengthening of the virtual AL bundle with increasing knee flexion. Based on their findings, the PM bundle was generally lax throughout the studied range of flexion and had a shortening phase up to 40°- 60° of flexion followed by an elongation trend thereafter. Garbelotti and co-workers also found the length of the mid-PCL unchanged during the first 30° of flexion with an elongation phase thereafter. These results are not in agreement with those previously reported by Wang and Walker [90] who used lateral and anterior-posterior radiographs from 12 cadaveric knees to calculate the relative displacement of the mid-PCL attachment sites marked with metal pins. They found a 10% negative strain during the first 30° of knee flexion, which remained nearly constant throughout the higher flexion angles. Nakagawa et al. [32] used sagittal MR-images of six cadaveric knees to assess elongation of the mid-PCL. They reported a steady increase in the length of the mid-PCL bundle from full extension to 120° of passive flexion with a maximum of 26% VBS.

One common approach to assess the kinematics of the PCL has been based on superimposing or registering the geometrical data obtained by digitizing bone surfaces and ligament attachment sites onto captured kinematic data. To capture the joint kinematics, Trent and co-workers [61] took photographs of the cadaveric specimens surrounded by several mirrors. Repeating this procedure for different flexion angles, they reported the pattern of ligament lengthening during 105° of knee flexion. In the first 15° of flexion, the mid-PCL bundle experienced shortening, followed by lengthening at higher flexion angles. However, recent studies in this field have employed more advanced technologies to capture the joint kinematics including 3D scanners [19], surgical navigation systems [25], optical markers [82], and electrogoniometers [22, 27] etc. Using such equipment, the elongation patterns reported by Wang et al. [19], Zaffagnini et al. [22] and Cross et al. [25] suggest lengthening of both the AL and PM bundles throughout the whole range of flexion. Belvedere and co-workers [23] as well as Hsieh and Draganich [27] found similar elongation patterns for the AL bundle but they reported a phase of shortening for the PM bundle starting from full extension. The results of Amiri and co-workers [82], however, suggest a very different loading pattern for the PCL, with negative strains for both bundles over the entire arc of flexion.

#### Modelling results

Seven studies used modelling approaches to calculate strain patterns of the PCL during passive knee flexion. As a simple method, four-bar linkage mechanisms formed by the tibia, the femur and the cruciate ligaments were used to simulate passive motion of the tibio-femoral joint and investigate ligament kinematics. Using a 2D linkage model of the knee, Zavatsky and O’Connor [38] calculated strain patterns of different bundles within the PCL during passive knee flexion. Their results demonstrated that the length of the anterior bundle gradually increased with knee flexion until 90° and remained constant thereafter, but the posterior bundle was positively strained only after 110° of flexion. Chittajallu and Kohrt [35] constructed a 2D dynamic model of the knee consisting of two coupled four-bar linkages driven by the cruciate and the collateral ligaments. Here, the governing equations of motion were solved to minimise the generated anterior-posterior force in the joint. The results indicated that until 60° of flexion, the mid-PCL strain had a gentle increase with a maximum of 1% strain but with a declining trend thereafter. Crowninshield and co-workers [34] introduced a 3D kinematic model of the knee that was driven by the motion of the joint centres of rotation and calculated negative strain patterns for both the anterior and posterior bundles throughout the whole range of flexion. Taking into account the contact between tibia and femur, Beynnon and co-workers [75], Wismans and co-workers [92] as well as Amiri and co-workers [84] developed quasi-static analytical models of the knee with bones represented as rigid bodies and ligaments as flexible one-dimensional elements. Although in these studies joint flexion was consistently achieved by applying a small force to the femur, the obtained strain patterns of the PCL bundles were not consistent. For instance, Wismans and co-workers [92] reported mid-PCL load-bearing only after approximately 40° of flexion but Amiri and co-workers found it tense only up to 50° of flexion.

**Fig 2:**
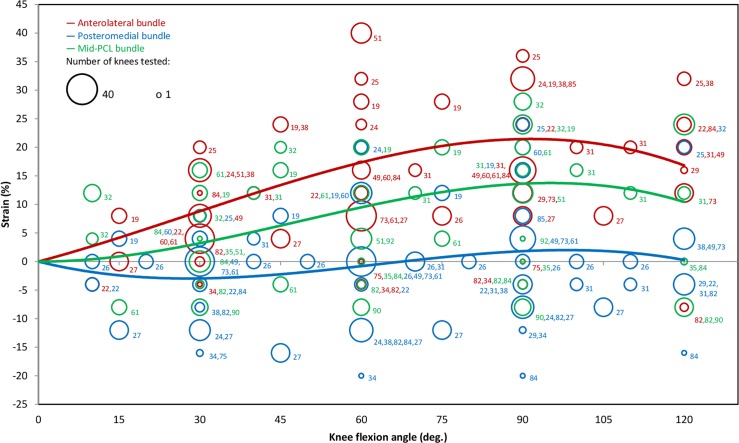
Binned scatter plot and weighted polynomial regression lines of all reported strain data for the AL, PM and mid-PCL bundles during passive knee flexion (R^2^ of 0.68, 0.04 and 0.46 respectively). Each circle shows the mean of the data extracted from the individual studies, where the size of the circle represents the number of knees tested and the numerical labels detail the reference numbers from which data were extracted. Data points with strains of less than -25% or greater than +45% are not shown (resolution of bins: ±2%).

In general, considerable variation was observed across the literature for the strain estimations within the AL, PM and mid-PM bundles (Fig 2). Nearly all studies suggested a positive strain in the AL bundle throughout the range of flexion, while both positive and negative strains were frequently reported for the PM bundle. As a result, the weighted-regression line for the AL bundle showed the highest strain magnitudes. The data indicate a gradual increase in AL bundle strain up to 90°, with a maximum of 21%, after which it declines to 17% at 120° of flexion. The regression line for the mid-PCL strain suggests a similar behaviour to that of the AL bundle but with lower magnitudes, and with a maximum of 14% at 90°. However, the weighted regression trend for the PM bundle strain indicates a slightly different pattern from those of the AL and mid-PCL bundles. In the first 80° of flexion, the bundle is shorter than its reference length, suggesting a lax state. The bundle has its shortest length at 30° joint flexion, with a corresponding strain of -3%. At 100°, the PM bundle presents its maximum positive strain of only 2%, and further flexion reduces the strain once again.

#### Difference between assessment techniques

**Fig 3:**
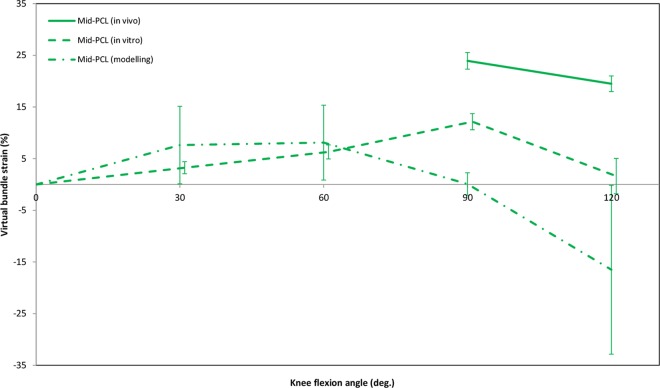
Strain of the virtual mid-PCL bundle during passive knee flexion, separated according to the assessment categories used. Articles in this graph included all data available for the mid-PCL: *in vivo* studies [31, 32], *in vitro* studies [19, 32, 51, 61, 82, 90], and modelling studies [35, 84, 92]. Experimental data were weighted based on the number of knees tested. Error bars show the Standard Error of Mean.

**Fig 4:**
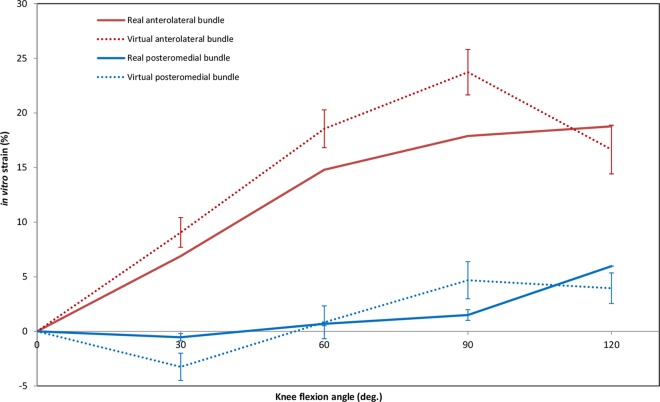
Strain of the real and virtual bundles of the PCL during passive knee flexion. Data were weighted based on the number of knees tested. Articles included in this graph: Studies on real bundles [26, 49] and studies on virtual bundles [19, 22, 24, 25, 27, 29, 51, 60, 61, 73, 82].

Average strain patterns obtained for the virtual mid-PCL bundle varied among the different assessment techniques (Fig 3). No *in vivo* strain data were reported for bundles at 30° or 60° of flexion. At 90°, modelling studies calculated mostly negligible strains (0%) for the mid-PCL, while *in vivo* investigations found mean strains of 24%; approximately twice the values obtained *in vitro*. However, all the assessment techniques suggested a declining trend for the bundle strain above 90° of knee flexion. Compared to *in vivo* and *in vitro* investigations, strain data estimated by means of modelling techniques had considerably higher variation. Mean VBS and RBS patterns exhibited similar trends (Fig 4). Both direct and indirect measurement techniques reported elongation of the AL bundle throughout the first 90°, and shortening of the PM bundle during the first 30° of flexion. Contrary to direct methods, however, indirect methods of strain measurement showed a declining strain after 90° of flexion for both bundles. Moreover, the strain magnitudes of the AL and PM bundles were found to exhibit different patterns throughout flexion, with considerably higher strains reported for the AL bundle.

### Force Patterns of the PCL during Passive Flexion

**Fig 5:**
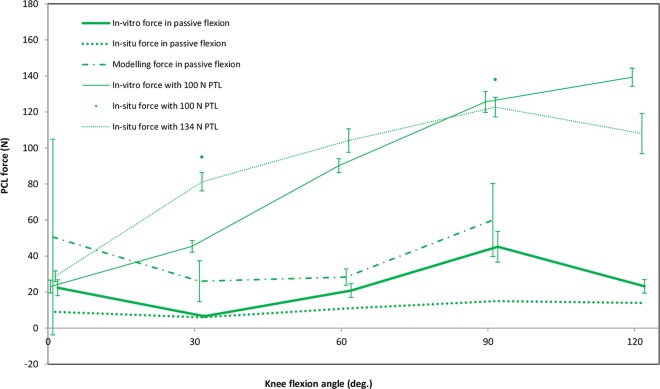
Average force patterns of the PCL during knee flexion with and without Posterior Tibial Load (PTL). Included articles in this graph: *in vitro* force in passive flexion [12, 13, 20, 62, 63, 86–89, 91], *in situ* force in passive flexion [9], modelling force in passive flexion [36, 75, 84], *in vitro* force with 100 N PTL [12, 13, 20, 86–88, 96, 97], *in situ* force with 100 N PTL [17] and *in situ* force with 134 N PTL [8, 15, 16, 18, 93–95, 98, 105].

The majority of force data reported for the PCL during passive flexion has been acquired by the same research group using cadaveric knees and attaching a load cell to the isolated femoral attachment site of the ligament [12, 13, 20, 62, 63, 86–89]. In these studies, the knees were mounted in multi-degree of freedom jigs and manually moved through knee flexion. Based on their measurements, the PCL was found to be under low tension throughout flexion. The average tension in the PCL at full extension, 30° and 120° of flexion was approximately 15 (3), 4 (1) and 19 (5)N respectively (Fig 5). Smaller forces were reported by Wang and co-workers [91] who used a similar assessment technique, and were in a general agreement with a study using buckle force transducers implanted on the PM bundle [63]. Miyasaka and co-workers [62] detached the PCL from its femoral attachment site and reattached it to its anatomical position using a metal plate instrumented with 12 strain gauges. They reported only small forces in the PCL throughout the range of passive flexion with a maximum of 4 (1)N at 90°. In another study, a robotic manipulator together with a universal force sensor (UFS) was used to measure the *in situ* force of the PCL in nine cadaveric knees during passive flexion [9]. Except for 90° flexion, the force patterns of the PCL were close to the average force trend extracted from the data measured *in vitro*.

Three studies presented modelling techniques to calculate the resultant PCL force during simulated passive flexion of the knee (Fig 5). With a 3D quasi-static model, Amiri and co-workers [84] found the PCL to be slack at full extension, but the model predicted an increase in the ligament force to 40N at 30° that remained nearly unchanged until 90° of flexion. However, between 90° and 120° of flexion, a dramatic increase to 290N was calculated. Different results were achieved using a 3D finite element model of the knee [36], where the PCL was reported to become load-bearing only after 20° of flexion, and a maximum of 35N was predicted above 90° flexion. Contrary to these studies, large tensions in both PCL bundles at full extension were calculated using a 2D quasi-static model of the knee [75]. Although the average force trend extracted from modelling studies had a similar pattern to that obtained from *in vitro* cadaveric investigations, analytical models generally overestimated the PCL tension at all flexion angles.

PCL forces in the presence of a posterior tibial load (PTL) have been examined using load cells attached to the isolated femoral bony attachment of the PCL in cadaveric knees [12, 13, 20, 86–89, 96, 97]. These studies indicated that the PCL is subjected to approximately 22N tensile force at full extension, with a steady increase to 128N at 90° of flexion (Fig 5), followed by only a small increase (approximately 10N) thereafter. These values were corroborated by Vahey and Draganich [17] using an indirect measurement technique, who estimated *in situ* forces 95N and 138N at 30° and 90° of flexion, respectively. In the presence of a 134N PTL, the PCL *in situ* force has also been indirectly assessed using robotic manipulators together with universal force sensors [8, 15, 16, 18, 93–95, 98]. These investigations indicated a relatively small force (29N) at full extension that gradually increased to 123N at 90° of flexion, and that reduced slightly thereafter. In general, the application of PTLs produced higher PCL forces than passive flexion, with the greatest difference at mid-flexion.

### Strain Patterns of the PCL during Active Flexion

**Fig 6:**
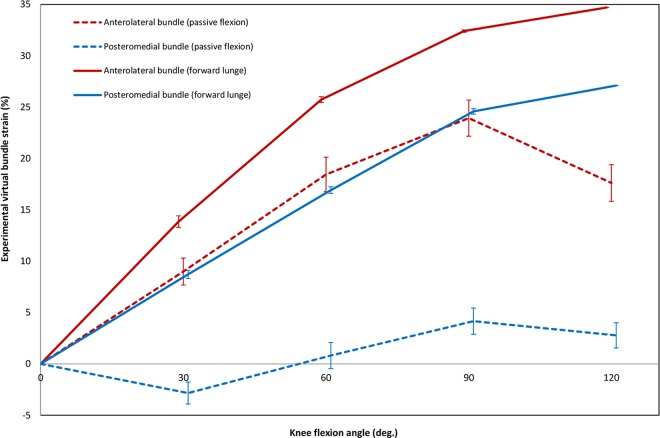
Average experimental strain patterns of the virtual PCL bundles during passive and active knee flexion. Included articles in this graph: Studies on passive flexion [19, 22, 24, 25, 27, 29, 51, 60, 61, 73, 82] and studies on forward lunge [14, 30, 33, 57].

All studies examining forward lunge or squat used image-based techniques for strain measurement. To analyse joint kinematics during forward lunge, 3D geometrical models of the subjects’ knees were registered to orthogonal fluoroscopic images captured at different knee flexion angles [14, 30, 33, 57]. Lengths of the virtual bundles were estimated by tracking the reconstructed motion of the ligament attachment sites, reporting an increase in AL, PM and mid-PCL VBSs throughout flexion (Fig 6).

At 90° of flexion, the average VBS in the AL and PM bundles were 32% and 25%. Surprisingly, the corresponding value for the mid-PCL was reported as being outside the values presented for the AL and PM bundles at approximately 23%. At 120° flexion, VBS for AL and PM bundles were reported as 35% and 27% respectively [14]. A comparison between the experimental VBS patterns of the PCL bundles during forward lunge (loaded) with those of passive flexion (unloaded) revealed a considerable difference in the mean strains that became larger at higher flexion angles (Fig 6). Furthermore, no relaxation phase for the PM bundle could be observed during forward lunge. Moreover, contrary to passive knee flexion, the strain magnitudes of the PCL during forward lunge did not decline after 90° of flexion.

For body-weight squat, the only study that contained strain data was an *in vivo* investigation by Nakagawa et al. [32], who included MR-imaging of the knees in thirteen healthy subjects. They found a consistent elongation for the mid-PCL bundle up to 31% at 120°, followed by a declining trend. At 90° of flexion, the average VBS for the mid-PCL was calculated as 28%, which was somewhat higher than the corresponding value for forward lunge (23%) reported by Defrate and co-workers [30].

### Force Patterns of the PCL during Active Flexion

Using a 2D musculoskeletal model, Shelburne and Pandy [99] calculated the PCL force up to 90° flexion during squats. The model predicted zero PCL force until 10° of knee flexion but a steadily increasing force thereafter to a peak of 650N at 80°. The estimated PCL force decreased slightly between 80° and 90° flexion. Using similar approaches, Toutoungi and co-workers [47] reported comparably small forces in the PCL during early knee flexion but predicted a much larger maximum PCL force of 2432N at 100° compared to Shelburne and Pandy [99]. Utilizing a two-stage procedure, Shelburne and co-workers [37] reported peak PCL forces of 274N during squatting, suggesting inconsistent results using musculoskeletal modelling approaches.

### Strain and Force Patterns of the PCL during Walking and Stair Activities

All studies reporting PCL load data during walking used modelling techniques, but with highly variable results (Table 4) [37, 44, 77, 78, 100–104]. While some investigators calculated almost no tension in the PCL (ranging from 0 to 27N) [37, 44, 104], others found relatively high maximum PCL forces (0.2–1 times body-weight; BW) [77, 78, 100–102]. The time-point at which the peak PCL force occurred was also inconsistent between different studies and varied from early stance to late swing phase. Two studies reported on the strain patterns of the PCL during stair ascent or stair descent *in vitro*. Mahoney and co-workers [71] measured *in vitro* strain in the AL bundle using implanted HEST sensors while the knee specimens were loaded to simulate stair ascent and descent. Until 40° of flexion, no significant strain was detected within the PCL for either activity studied. During stair ascent, the maximum RBS in the AL bundle was measured as 3%, which occurred between 100° and 110° flexion. Similarly, during stair descent, peak strains of 3% were measured between 100° and 110°, but these results were somewhat lower than the results of Emodi and co-workers [58] who reported strains of 9% in the AL bundle at 105° flexion during stair descent using implanted differential variable reluctance transducer (DVRT) strain sensors.

## Discussion

The PCL is known to be one of the four major stabilizers of the knee joint. Many aspects of PCL biomechanics have been investigated based on strain and force measures of the ligament during different activities. However, due to the high variability in the reported load data, controversy still surrounds the definitive role of the ligament and its functional bundles during normal daily activities. In this comprehensive analysis of the literature, new understanding of the force and strain patterns within the PCL of healthy knees have been gained that would not be possible from a single study alone. Here, we have extracted the mean strain and force patterns of the PCL from an initial screening of over 3500 scientific articles, to provide the foundations for understanding PCL loading patterns during different activities. In addition, the influence of different assessment techniques on variations of the reported load data has been investigated as a possible source of bias.

This systematic review has revealed that current knowledge of PCL loading mainly suffers from an insufficient number of studies, particularly during active and deep flexion activities. For example, no *in vivo* sensor-based study exists that have measured PCL force or strain in healthy human subjects. Only few studies have reported lengthening patterns of the virtual PCL bundles *in vivo* using indirect assessment techniques; and these studies have been conducted by single research groups or were limited to quasi-static forms of passive flexion, body-weight squat or forward lunge. *In vitro* cadaveric investigations on PCL loading are more frequent, but the majority have assessed PCL loads using indirect methods. While some *in vitro* studies have utilised strain or force sensors to investigate the effects of externally applied loads or muscle contractions on the ligament load, only a few of them have accurately captured the physiological loading conditions. Information on PCL loading from musculoskeletal and finite element modelling studies is limited and seems to be highly variable. As such, this comprehensive systematic review of the literature represents the single best understanding of loading conditions within the PCL available. Based on the statistical analysis of the reported data, the loading conditions in the PCL and its two main functional bundles during passive flexion have now been characterised in detail (Fig 2). However, studies on PCL biomechanics during physiological activities of daily living, as well as the extreme conditions experienced during impact or intense sport, are clearly missing and need additional investigation.

The average loading patterns of the PCL confirm that both PCL strain and force magnitudes are dependent upon the knee flexion angle. During passive flexion, strains of the virtual AL and mid-PCL bundles follow similar patterns, consisting of an upward trend in the first 90° and a declining trend thereafter. With the zero strain condition defined at full extension of the knee, the PM bundle remains relatively relaxed throughout flexion. Interestingly, the magnitudes of the mid-PCL strains were between those of the AL and PM bundles. This fact adds confidence to the performed statistical analysis as the strain data were almost entirely extracted from independent studies. Although VBS does not seem to decline after 90° of flexion during forward lunges or squats, the slopes of the average strain patterns decreased at higher flexion angles. It was also observed that a posterior tibial load affects the PCL force more notably in the mid-range of knee flexion compared to early or deep flexion (c.f. *in situ* force passive *vs* 134N PTL; Fig 5), due to the reduced restraining function of the PCL at low and high flexion angles. Although we did not show PCL loading beyond 120° in this review, Li and co-workers [106] reported only a small contribution of the PCL to knee stability at 150° of flexion. This behaviour can be partially explained by changes in the spatial orientation of the PCL bundles, which become more vertically oriented at higher knee flexion angles, and therefore lose their efficiency in restraining posterior shear forces [14, 22, 30, 32, 33]. However, some studies have reported an increasing trend for the posterior shear force acting on the knee joint throughout the whole range of knee flexion [107, 108]. This raises the question of which structures compensate for the weak restraining function of the PCL against posterior tibial loads at higher knee flexion angles. Here, posterior structures of the knee including skin, fat, hamstring muscles, joint capsule and meniscus are thought to partially provide posterior stability to a hyper-flexed knee joint, even though their individual contributions remain unknown [106].

It is known that knee kinematics are activity-specific [109], suggesting that PCL strain is also affected by activity type [31]. Our study supports this idea as the extracted strain patterns of the ligament bundles differ between passive flexion and forward lunge (Fig 6). This difference, which is more notable at higher knee flexion angles, might originate from variations in the contributions of muscle forces to knee joint kinematics during different activities. Both the quadriceps and the hamstrings muscle groups are involved during a forward lunge [110]. While, contraction of the quadriceps can lead to a slight decrease of the PCL force, contraction of the hamstrings has been shown to considerably increase the PCL load [86]. Therefore, compared to passive knee flexion, it is entirely plausible that the PCL is exposed to higher loads during sports activities.

Variations in the reported strain and force data of the PCL during physiological loading might originate from different sources. The anatomy of the PCL is subject-specific, and geometrical parameters of the ligament including length, cross sectional area and attachment sites of the bundles have been described inconsistently [39, 60]. Therefore, it is likely that ligament strain varies among different subjects performing the same activity. It is also known that the lengthening pattern of the PCL is sensitive to the definition of its attachment sites, which is likely accompanied by errors especially when image-based techniques are used [111]. In case of cadaveric investigations, specimens were prepared in different ways depending on the type and volume of the surrounding soft tissues resected. Furthermore, joint kinematics was simulated using a variety of techniques including manual, mechanical and robotic approaches, as well as internal or external poses of the bones, possibly adding to the reported variation in outcome measures. Additionally, strain of the ligament was measured using different direct and indirect methodologies. Given the many techniques and methods employed in assessing PCL loading, it is therefore hardly surprising that the reported outcomes vary considerably between the different studies.

This systematic review provided the opportunity to compare RBS of the PCL (via direct approaches) with VBS (via indirect approaches) during passive knee flexion. The results from the statistical analysis demonstrate that VBS patterns during passive knee flexion are similar to RBS patterns but differ in magnitude. This should be kept in mind when interpreting results from studies reporting VBS; in particular, large VBS magnitudes may falsely imply failure of a ligament that in reality experiences safe strain magnitudes. For example, at 90° of passive flexion, the PCL was shown to be very lightly loaded (Fig 5); however, the average experimental VBS of the AL bundle was calculated at 24%, which is higher than the failure strain measured *in situ* (18%) [41]. Surprisingly, the calculated maximum VBS during forward lunge (35%) is approximately twice the magnitude of the bundle failure strain. The difference between VBS and RBS likely originates from the unknown zero-strain condition. Given the technical difficulties in identifying the accurate zero-strain condition, nearly all the included studies chose full extension of the knee as an arbitrary reference position, while in reality the PCL is considered lax at this position [6, 24]. Therefore, the selection of the reference position may add a systematic error to the strain data obtained by measuring the relative elongation of the virtual bundles. Moreover, in cases where the ligament is lax or wraps around a neighbouring structure, the straight-line representation between the attachment sites–or “virtual bundle”–does not represent the curvilinear length of the real bundle. Another key factor that might intensify the difference between RBS and VBS (especially for ligaments with large cross sectional areas) is the distribution of the strain across the ligament. RBS is generally measured using sensors attached to the surface of the ligament and therefore represents the local superficial strain. In contrast, VBS tends to represent the average strain of a virtual fibre inside the ligament. King and co-workers [31] showed that large strain differences exist between the superficial fibres and the virtual lines that connect the attachment sites. While VBS can be used for intra-study comparison of the ligament strains under different loading conditions, any inter-study comparison of VBS is limited to investigations that have used a common reference length.

Given the difficulties in interpreting ligament strain data from different studies, ligament force may be a more useful measure for investigating PCL biomechanics, especially considering the role of ligament viscoelastic properties in modulating the force—elongation relationships. PCL force can be measured directly by implanting force sensors; however, current sensors are somewhat large and stiff compared to the PCL structure and therefore interfere with normal ligament function. An alternative approach includes the use of robotic technology to measure the *in situ* force of the ligament based on the principle of superposition, thus avoiding limitations induced by direct contact of the force sensor and ligament. In this review, only a small difference was found between the *in vitro* and *in situ* forces determined in the PCL during passive knee flexion. In general, the PCL was found to be very slightly tense throughout flexion, with the smallest tension at 30° of knee flexion. Applying a PTL was shown to increase the PCL force at all flexion angles, but the increase was more considerable at mid-range of knee flexion. The fact that a PTL does not strongly affect the PCL force at full extension may explain why an isolated rupture of the PCL does not often lead to knee instability. Previous studies confirmed the role of meniscofemoral ligaments as well as posterolateral and posteromedial structures of the knee in providing knee stability near full knee extension [5, 112]. In the majority of reported studies, PTL had the greatest effect on the PCL force at 90° of knee flexion. This could explain the so called “dashboard injury” as the most common mechanism of PCL injury [55]. During a traffic accident, the knee hits the dashboard while it is at approximately 90° of flexion and the posteriorly directed impact force tears the ligament. This finding is in agreement with studies reporting that an isolated sectioning of the PCL causes the highest change in posterior tibial translation in response to an applied PTL at 90° of flexion [1, 8]. Therefore, clinical testing for the integrity of the ligament should be performed at this position to obtain the highest sensitivity.

We found that the choice of assessment technique affects the resulting loading patterns in the PCL. Ideally, PCL force or strain would be measured directly *in vivo* using implantable sensors. However, such *in vivo* procedures are generally invasive and the outcomes are limited to relative strain at the implantation site. Moreover, the imposed size and stiffness of the available sensors interfere with the natural ligament kinematics. Indirect assessment of the ligament strain via image-based tracking of the ligament attachment sites would therefore seem to deserve more attention for *in vivo* investigation of the ligament strain. Despite using substantially different methodologies, the mean strain patterns extracted from data obtained using indirect techniques follow those of direct investigations. This finding confirms the ability of indirect techniques to estimate the ligament strains. However, a correlation study to determine the relationship between RBS and VBS during different activities, and based on an adequate number of specimens tested, appears to be missing. Cadaveric investigations have provided the major contribution to our current knowledge on PCL loading; however, physiological loading conditions are difficult to reproduce in mechanical jigs. Finite element and musculoskeletal modelling techniques allow the simulation of different loading conditions [113], but limitations remain with respect to the accurate definition of the geometry, and the material properties, as well as the contact conditions within the joint and specifically with the ligament. As a consequence, compared to the load data measured *in vivo* or *in vitro*, the highest reported variability in the load data was obtained using modelling techniques. This fact emphasises the need to account for the 3D geometry, viscoelastic material properties and wrapping of the ligaments around neighbouring structures in future modelling studies.

Some limitations of this systematic review need to be recognized. Only a limited number of activities were investigated, and also within a limited range of flexion (0° to 120°). Due to a lack of reported strain and force data, the loading patterns of the ligament during hyperextension and hyperflexion of the knee were not assessed in this review. In addition to a shortage of studies, the various assessment techniques used for measuring the ligament loading, in combination with the large number of dependent variables (flexion angle, bundles, activity etc.), preclude an effective statistical comparison to reveal the role of individual parameters on PCL loading. In some studies, the standard deviations in the reported outcome measures were not given, while in some others, the data were difficult to extract from e.g. graphical presentations. Consequently, standard deviations could only partially be taken into account for proper weighting of data points in the present statistical analysis. Many of the included studies were performed by the same research groups; a fact that might increase the risk of bias. Finally, while we have investigated PCL loading in the healthy knee joint, PCL function and its relationship with loading and kinematics in pathological cases is still less well understood.

Given the insight obtained from this review, it is clear that certain considerations could help improve comparability of future investigations towards understanding PCL force and strain patterns. We propose that future studies should:

- Adopt a standard definition of PCL anatomy, including the anterolateral and posteromedial bundles [2].
- Use a standardised knee joint coordinate system [114] for reporting the simulated bone kinematics.
- Clearly report the approach taken to define zero-strain position.
- Use standardised terminology (consistent with this review) for presenting results.
- Couple *in vivo* image-based studies with *in vitro* sensor-based studies to relate VBS and RBS.

## Conclusions

This systematic review has provided an overview of the content and limitations in the current knowledge on PCL loading in the healthy knee. In general, the results of this review confirm that the individual bundles within the PCL are subjected to different loading patterns that are affected by both activity type and knee flexion angle. Moreover, it was found that the choice in assessment technique might have an important effect on the resulting load data. In the context of injury prevention, the extracted strain and force trends now provide the foundations for improved risk of injury assessment. The extracted loading patterns can also help determine the critical positions of the knee joint at which clinical tests are more sensitive to ligament deficiencies. In the context of PCL reconstruction, efforts should focus on restoring the loading patterns of the healthy ligament as summarised in this review. Finally, for rehabilitation of PCL injury, the range of knee joint motion during prescribed exercises can be adjusted based on safe strain levels to prevent any possible rupture of the reconstructed ligament.

## Supporting Information

S1 File Search string used for the review. (DOC)

S2 File PRISMA checklist. (DOC)

## References

[1] Kennedy NI, Wijdicks CA, Goldsmith MT, Michalski MP, Devitt BM, Aroen A, et al Kinematic analysis of the posterior cruciate ligament, part 1: the individual and collective function of the anterolateral and posteromedial bundles. The American journal of sports medicine. 2013;41(12):2828–38. 10.1177/0363546513504287 . 24064797

[2] LaPrade CM, Civitarese DM, Rasmussen MT, LaPrade RF. Emerging Updates on the Posterior Cruciate Ligament: A Review of the Current Literature. The American journal of sports medicine. 2015 10.1177/0363546515572770 . 25776184

[3] Wang CJ. Injuries to the posterior cruciate ligament and posterolateral instabilities of the knee. Chang Gung medical journal. 2002;25(5):288–97. . 12141701

[4] Logan M, Williams A, Lavelle J, Gedroyc W, Freeman M. The effect of posterior cruciate ligament deficiency on knee kinematics. The American journal of sports medicine. 2004;32(8):1915–22. . 15572321 10.1177/0363546504265005

[5] Amis AA, Bull AMJ, Gupte CM, Hijazi I, Race A, Robinson JR. Biomechanics of the PCL and related structures: posterolateral, posteromedial and meniscofemoral ligaments. Knee Surg Sport Tr A. 2003;11(5):271–81. . 10.1007/s00167-003-0410-7 12961064

[6] Girgis FG, Marshall JL, Monajem A. The cruciate ligaments of the knee joint. Anatomical, functional and experimental analysis. Clin Orthop Relat Res. 1975;(106):216–31. . 1126079 10.1097/00003086-197501000-00033

[7] Grood ES, Noyes FR, Butler DL, Suntay WJ. Ligamentous and capsular restraints preventing straight medial and lateral laxity in intact human cadaver knees. The Journal of bone and joint surgery American volume. 1981;63(8):1257–69. . 7287796

[8] Harner CD, Janaushek MA, Kanamori A, Yagi M, Vogrin TM, Woo SL. Biomechanical analysis of a double-bundle posterior cruciate ligament reconstruction. The American journal of sports medicine. 2000;28(2):144–51. . 10750988 10.1177/03635465000280020201

[9] Hoher J, Vogrin TM, Woo SL, Carlin GJ, Aroen A, Harner CD. In situ forces in the human posterior cruciate ligament in response to muscle loads: a cadaveric study. Journal of orthopaedic research: official publication of the Orthopaedic Research Society. 1999;17(5):763–8. 10.1002/jor.1100170522 . 10569489

[10] Kurosawa H, Yamakoshi K, Yasuda K, Sasaki T. Simultaneous Measurement of Changes in Length of the Cruciate Ligaments during Knee Motion. Clin Orthop Relat R. 1991;(265):233–40. . 2009664

[11] Li G, Papannagari R, Li M, Bingham J, Nha KW, Allred D, et al Effect of posterior cruciate ligament deficiency on in vivo translation and rotation of the knee during weightbearing flexion. Am J Sport Med. 2008;36(3):474–9. . 10.1177/0363546507310075 18057390

[12] Markolf KL, Jackson SR, McAllister DR. Single- versus double-bundle posterior cruciate ligament reconstruction: effects of femoral tunnel separation. The American journal of sports medicine. 2010;38(6):1141–6. 10.1177/0363546509359072 . 20348284

[13] Markolf KL, Slauterbeck JR, Armstrong KL, Shapiro MS, Finerman GA. A biomechanical study of replacement of the posterior cruciate ligament with a graft. Part II: Forces in the graft compared with forces in the intact ligament. The Journal of bone and joint surgery American volume. 1997;79(3):381–6. . 9070527 10.2106/00004623-199703000-00010

[14] Papannagari R, DeFrate LE, Nha KW, Moses JM, Moussa M, Gill TJ, et al Function of posterior cruciate ligament bundles during in vivo knee flexion. The American journal of sports medicine. 2007;35(9):1507–12. 10.1177/0363546507300061 . 17376856

[15] Petersen W, Lenschow S, Weimann A, Strobel MJ, Raschke MJ, Zantop T. Importance of femoral tunnel placement in double-bundle posterior cruciate ligament reconstruction: biomechanical analysis using a robotic/universal force-moment sensor testing system. The American journal of sports medicine. 2006;34(3):456–63. 10.1177/0363546505281239 . 16303880

[16] Sekiya JK, Haemmerle MJ, Stabile KJ, Vogrin TM, Harner CD. Biomechanical analysis of a combined double-bundle posterior cruciate ligament and posterolateral corner reconstruction. The American journal of sports medicine. 2005;33(3):360–9. . 15716251 10.1177/0363546504268039

[17] Vahey JW, Draganich LF. Tensions in the anterior and posterior cruciate ligaments of the knee during passive loading: predicting ligament loads from in situ measurements. Journal of orthopaedic research: official publication of the Orthopaedic Research Society. 1991;9(4):529–38. 10.1002/jor.1100090408 . 2045979

[18] Vogrin TM, Hoher J, Aroen A, Woo SL, Harner CD. Effects of sectioning the posterolateral structures on knee kinematics and in situ forces in the posterior cruciate ligament. Knee surgery, sports traumatology, arthroscopy: official journal of the ESSKA. 2000;8(2):93–8. 10.1007/s001670050193 . 10795671

[19] Wang JH, Kato Y, Ingham SJ, Maeyama A, Linde-Rosen M, Smolinski P, et al Effects of knee flexion angle and loading conditions on the end-to-end distance of the posterior cruciate ligament: a comparison of the roles of the anterolateral and posteromedial bundles. The American journal of sports medicine. 2014;42(12):2972–8. 10.1177/0363546514552182 . 25315993

[20] Wascher DC, Markolf KL, Shapiro MS, Finerman GA. Direct in vitro measurement of forces in the cruciate ligaments. Part I: The effect of multiplane loading in the intact knee. The Journal of bone and joint surgery American volume. 1993;75(3):377–86. . 8444916 10.2106/00004623-199303000-00009

[21] Wunschel M, Leasure JM, Dalheimer P, Kraft N, Wulker N, Muller O. Differences in knee joint kinematics and forces after posterior cruciate retaining and stabilized total knee arthroplasty. Knee. 2013;20(6):416–21. 10.1016/j.knee.2013.03.005 . 23578828

[22] Zaffagnini S, Martelli S, Garcia L, Visani A. Computer analysis of PCL fibres during range of motion. Knee surgery, sports traumatology, arthroscopy: official journal of the ESSKA. 2004;12(5):420–8. 10.1007/s00167-004-0502-z . 15060763

[23] Belvedere C, Ensini A, Feliciangeli A, Cenni F, D'Angeli V, Giannini S, et al Geometrical changes of knee ligaments and patellar tendon during passive flexion. Journal of biomechanics. 2012;45(11):1886–92. 10.1016/j.jbiomech.2012.05.029 . 22677336

[24] Blankevoort L, Huiskes R, de Lange A. Recruitment of knee joint ligaments. Journal of biomechanical engineering. 1991;113(1):94–103. . 2020181 10.1115/1.2894090

[25] Cross MB, Raphael BS, Maak TG, Plaskos C, Egidy CC, Pearle AD. Characterization of the orientation and isometry of Humphrey's ligament. Knee. 2013;20(6):515–9. 10.1016/j.knee.2013.04.002 . 23659994

[26] Dürselen L, Claes L, Kiefer H. The influence of muscle forces and external loads on cruciate ligament strain. The American journal of sports medicine. 1995;23(1):129–36. . 7726343 10.1177/036354659502300122

[27] Hsieh YF, Draganich LF. Knee kinematics and ligament lengths during physiologic levels of isometric quadriceps loads. Knee. 1997;4(3):145–54. .

[28] Markolf KL, Feeley BT, Jackson SR, McAllister DR. Biomechanical studies of double-bundle posterior cruciate ligament reconstructions. The Journal of bone and joint surgery American volume. 2006;88(8):1788–94. 10.2106/JBJS.E.00427 . 16882903

[29] van Dijk R, Huiskes R, Selvik G. Roentgen stereophotogrammetric methods for the evaluation of the three dimensional kinematic behaviour and cruciate ligament length patterns of the human knee joint. Journal of biomechanics. 1979;12(9):727–31. . 489639 10.1016/0021-9290(79)90021-6

[30] DeFrate LE, Gill TJ, Li G. In vivo function of the posterior cruciate ligament during weightbearing knee flexion. The American journal of sports medicine. 2004;32(8):1923–8. . 15572322 10.1177/0363546504264896

[31] King AJ, Deng Q, Tyson R, Sharp JC, Matwiy J, Tomanek B, et al In vivo open-bore MRI reveals region- and sub-arc-specific lengthening of the unloaded human posterior cruciate ligament. PloS one. 2012;7(11):e48714 10.1371/journal.pone.0048714 23144939 PMC3492418

[32] Nakagawa S, Johal P, Pinskerova V, Komatsu T, Sosna A, Williams A, et al The posterior cruciate ligament during flexion of the normal knee. J Bone Joint Surg Br. 2004;86(3):450–6. . 15125137 10.1302/0301-620x.86b3.14330

[33] Yue B, Varadarajan KM, Rubash HE, Li G. In vivo function of posterior cruciate ligament before and after posterior cruciate ligament-retaining total knee arthroplasty. International orthopaedics. 2012;36(7):1387–92. 10.1007/s00264-011-1481-6 22270863 PMC3385884

[34] Crowninshield R, Pope MH, Johnson RJ. An analytical model of the knee. Journal of biomechanics. 1976;9(6):397–405. . 932053 10.1016/0021-9290(76)90117-2

[35] Chittajallu SK, and Kohrt K.G.. FORM2D—a mathematical model of the knee. Mathematical and Computer Modelling. 1996;24(9):91–101.

[36] Moglo KE, Shirazi-Adl A. Cruciate coupling and screw-home mechanism in passive knee joint during extension—flexion. Journal of biomechanics. 2005;38(5):1075–83. 10.1016/j.jbiomech.2004.05.033 . 15797589

[37] Shelburne KB, Kim HJ, Sterett WI, Pandy MG. Effect of posterior tibial slope on knee biomechanics during functional activity. Journal of orthopaedic research: official publication of the Orthopaedic Research Society. 2011;29(2):223–31. 10.1002/jor.21242 . 20857489

[38] Zavatsky AB, O'Connor JJ. A model of human knee ligaments in the sagittal plane. Part 2: Fibre recruitment under load. Proceedings of the Institution of Mechanical Engineers Part H, Journal of engineering in medicine. 1992;206(3):135–45. . 1482509 10.1243/PIME_PROC_1992_206_281_02

[39] Voos JE, Mauro CS, Wente T, Warren RF, Wickiewicz TL. Posterior cruciate ligament: anatomy, biomechanics, and outcomes. The American journal of sports medicine. 2012;40(1):222–31. 10.1177/0363546511416316 . 21803977

[40] Kennedy JC, Hawkins RJ, Willis RB, Danylchuck KD. Tension studies of human knee ligaments. Yield point, ultimate failure, and disruption of the cruciate and tibial collateral ligaments. The Journal of bone and joint surgery American volume. 1976;58(3):350–5. . 1262366

[41] Race A, Amis AA. The mechanical properties of the two bundles of the human posterior cruciate ligament. Journal of biomechanics. 1994;27(1):13–24. . 8106532 10.1016/0021-9290(94)90028-0

[42] Herzmark MH. The evolution of the knee joint. J Bone Joint Surg. 1938;20:77–84.

[43] Blacharski PA, Somerset JH, Murray DG. A three-dimensional study of the kinematics of the human knee. Journal of biomechanics. 1975;8(6):375–84. . 1206040 10.1016/0021-9290(75)90073-1

[44] Yang NH, Canavan PK, Nayeb-Hashemi H, Najafi B, Vaziri A. Protocol for constructing subject-specific biomechanical models of knee joint. Computer methods in biomechanics and biomedical engineering. 2010;13(5):589–603. 10.1080/10255840903389989 . 20521186

[45] Torg JS, Barton TM, Pavlov H, Stine R. Natural-History of the Posterior Cruciate Ligament-Deficient Knee. Clin Orthop Relat R. 1989;(246):208–16. 2766608

[46] Fowler PJ, Messieh SS. Isolated Posterior Cruciate Ligament Injuries in Athletes. Am J Sport Med. 1987;15(6):553–7. . 10.1177/036354658701500606 3425783

[47] Toutoungi DE, Lu TW, Leardini A, Catani F, O'Connor JJ. Cruciate ligament forces in the human knee during rehabilitation exercises. Clinical biomechanics. 2000;15(3):176–87. . 10656979 10.1016/s0268-0033(99)00063-7

[48] Escamilla RF, Fleisig GS, Zheng N, Barrentine SW, Wilk KE, Andrews JR. Biomechanics of the knee during closed kinetic chain and open kinetic chain exercises. Medicine and science in sports and exercise. 1998;30(4):556–69. . 9565938 10.1097/00005768-199804000-00014

[49] Arms SW, Johnson, R.J., Pope, M.H. Strain measurement of the human posterior cruciate ligament. 30th annual ORS, Atlanta, Georgia. 1984:355.

[50] Van Dommelen BA, Fowler PJ. Anatomy of the posterior cruciate ligament. A review. The American journal of sports medicine. 1989;17(1):24–9. . 2648873 10.1177/036354658901700104

[51] Garbelotti Junior SA, Pelozo Junior O, Caldana RP, Ramalho A Jr, Smith RL. Experimental evaluation of 3-dimensional kinematic behavior of the cruciate ligaments. Clinics. 2007;62(5):619–26. . 17952324 10.1590/s1807-59322007000500014

[52] Shelbourne KD, Davis TJ, Patel DV. The natural history of acute, isolated, nonoperatively treated posterior cruciate ligament injuries. A prospective study. The American journal of sports medicine. 1999;27(3):276–83. . 10352760 10.1177/03635465990270030201

[53] Miyasaka KC, Daniel D. M., Stone M. L., & Hirshman P. The incidence of knee ligament injuries in the general population. Am J Knee Surg. 1991;4(1):3–8.

[54] Fanelli GC, Edson CJ. Posterior cruciate ligament injuries in trauma patients: Part II. Arthroscopy: the journal of arthroscopic & related surgery: official publication of the Arthroscopy Association of North America and the International Arthroscopy Association. 1995;11(5):526–9. . 8534292 10.1016/0749-8063(95)90127-2

[55] Schulz MS, Russe K, Weiler A, Eichhorn HJ, Strobel MJ. Epidemiology of posterior cruciate ligament injuries. Archives of orthopaedic and trauma surgery. 2003;123(4):186–91. 10.1007/s00402-002-0471-y . 12734718

[56] Harner CD, Livesay GA, Kashiwaguchi S, Fujie H, Choi NY, Woo SL. Comparative study of the size and shape of human anterior and posterior cruciate ligaments. Journal of orthopaedic research: official publication of the Orthopaedic Research Society. 1995;13(3):429–34. 10.1002/jor.1100130317 . 7602404

[57] Li G, DeFrate LE, Sun H, Gill TJ. In vivo elongation of the anterior cruciate ligament and posterior cruciate ligament during knee flexion. The American journal of sports medicine. 2004;32(6):1415–20. 10.1177/0363546503262175 . 15310565

[58] Emodi GJ, Callaghan JJ, Pedersen DR, Brown TD. Posterior cruciate ligament function following total knee arthroplasty: the effect of joint line elevation. The Iowa orthopaedic journal. 1999;19:82–92. 10847521 PMC1888617

[59] Fox RJ, Harner CD, Sakane M, Carlin GJ, Woo SL. Determination of the in situ forces in the human posterior cruciate ligament using robotic technology. A cadaveric study. The American journal of sports medicine. 1998;26(3):395–401. . 9617402 10.1177/03635465980260030901

[60] Inderster A, Benedetto KP, Klestil T, Kunzel KH, Gaber O. Fiber orientation of posterior cruciate ligament: an experimental morphological and functional study, Part 2. Clinical anatomy. 1995;8(5):315–22. 10.1002/ca.980080502 . 8535962

[61] Trent PS, Walker PS, Wolf B. Ligament length patterns, strength, and rotational axes of the knee joint. Clin Orthop Relat Res. 1976;(117):263–70. . 1277674

[62] Miyasaka T, Matsumoto H, Suda Y, Otani T, Toyama Y. Coordination of the anterior and posterior cruciate ligaments in constraining the varus-valgus and internal-external rotatory instability of the knee. Journal of orthopaedic science: official journal of the Japanese Orthopaedic Association. 2002;7(3):348–53. 10.1007/s007760200058 . 12077660

[63] Lew WD, Lewis JL. The effect of knee-prosthesis geometry on cruciate ligament mechanics during flexion. The Journal of bone and joint surgery American volume. 1982;64(5):734–9. . 7085699

[64] Kohen RB, Sekiya JK. Single-bundle versus double-bundle posterior cruciate ligament reconstruction. Arthroscopy: the journal of arthroscopic & related surgery: official publication of the Arthroscopy Association of North America and the International Arthroscopy Association. 2009;25(12):1470–7. 10.1016/j.arthro.2008.11.006 . 19962075

[65] Kennedy JC, Hawkins RJ, Willis RB. Strain gauge analysis of knee ligaments. Clin Orthop Relat Res. 1977;(129):225–9. . 608281 10.1097/00003086-197711000-00031

[66] Li G, Zayontz S, Most E, Otterberg E, Sabbag K, Rubash HE. Cruciate-retaining and cruciate-substituting total knee arthroplasty: an in vitro comparison of the kinematics under muscle loads. The Journal of arthroplasty. 2001;16(8 Suppl 1):150–6. . 11742468 10.1054/arth.2001.28367

[67] Li G, Zayontz S, Most E, DeFrate LE, Suggs JF, Rubash HE. In situ forces of the anterior and posterior cruciate ligaments in high knee flexion: an in vitro investigation. Journal of orthopaedic research: official publication of the Orthopaedic Research Society. 2004;22(2):293–7. 10.1016/S0736-0266(03)00179-7 . 15013087

[68] Hinterwimmer S, Baumgart R, Plitz W. Strain measurement at the knee ligament insertion sites. Biomed Tech. 2003;48(1–2):11–4. . 10.1515/bmte.2003.48.1-2.11 12655843

[69] Fleming BC, Beynnon BD. In vivo measurement of ligament/tendon strains and forces: a review. Annals of biomedical engineering. 2004;32(3):318–28. . 15095807 10.1023/b:abme.0000017542.75080.86

[70] Rudy TW, Livesay GA, Woo SL, Fu FH. A combined robotic/universal force sensor approach to determine in situ forces of knee ligaments. Journal of biomechanics. 1996;29(10):1357–60. . 8884481 10.1016/0021-9290(96)00056-5

[71] Mahoney OM, Noble PC, Rhoads DD, Alexander JW, Tullos HS. Posterior cruciate function following total knee arthroplasty. A biomechanical study. The Journal of arthroplasty. 1994;9(6):569–78. . 7699369 10.1016/0883-5403(94)90110-4

[72] Ogata K, McCarthy JA, Dunlap J, Manske PR. Pathomechanics of posterior sag of the tibia in posterior cruciate deficient knees. An experimental study. The American journal of sports medicine. 1988;16(6):630–6. . 3239620 10.1177/036354658801600613

[73] Dorlot JM, Christel P, Sedel L, Witvoet J. The displacement of the bony insertion sites of the cruciate ligaments during the flexion of the knee. 29th Annual ORS, Anaheim, California. 1983:328.

[74] Ahmad CS, Cohen ZA, Levine WN, Gardner TR, Ateshian GA, Mow VC. Codominance of the individual posterior cruciate ligament bundles—An analysis of bundle lengths and orientation. Am J Sport Med. 2003;31(2):221–5. . 10.1177/03635465030310021101 12642256

[75] Beynnon B, Yu J, Huston D, Fleming B, Johnson R, Haugh L, et al A sagittal plane model of the knee and cruciate ligaments with application of a sensitivity analysis. Journal of biomechanical engineering. 1996;118(2):227–39. . 8738789 10.1115/1.2795965

[76] Thelen DG, Won Choi K, Schmitz AM. Co-simulation of neuromuscular dynamics and knee mechanics during human walking. Journal of biomechanical engineering. 2014;136(2):021033 10.1115/1.4026358 24390129 PMC4023657

[77] Collins JJ. The redundant nature of locomotor optimization laws. Journal of biomechanics. 1995;28(3):251–67. . 7730385 10.1016/0021-9290(94)00072-c

[78] Morrison JB. The mechanics of the knee joint in relation to normal walking. Journal of biomechanics. 1970;3(1):51–61. . 5521530 10.1016/0021-9290(70)90050-3

[79] Escamilla RF, Zheng N, Imamura R, Macleod TD, Edwards WB, Hreljac A, et al Cruciate ligament force during the wall squat and the one-leg squat. Medicine and science in sports and exercise. 2009;41(2):408–17. 10.1249/MSS.0b013e3181882c6d . 19127183

[80] Li G, Gil J, Kanamori A, Woo SL. A validated three-dimensional computational model of a human knee joint. Journal of biomechanical engineering. 1999;121(6):657–62. . 10633268 10.1115/1.2800871

[81] Pena E, Calvo B, Martinez MA, Doblare M. A three-dimensional finite element analysis of the combined behavior of ligaments and menisci in the healthy human knee joint. Journal of biomechanics. 2006;39(9):1686–701. 10.1016/j.jbiomech.2005.04.030 . 15993414

[82] Amiri S, Cooke TD, Wyss UP. A multiple-bundle model to characterize the mechanical behavior of the cruciate ligaments. Knee. 2011;18(1):34–41. 10.1016/j.knee.2010.01.003 . 20116260

[83] Thomas J, Brunton J., Graziosi S. EPPI-Reviewer 4: software for research synthesis EPPI-Centre Software. London: Social Science Research Unit, Institute of Education 2010.

[84] Amiri S, Cooke D, Kim IY, Wyss U. Mechanics of the passive knee joint. Part 2: interaction between the ligaments and the articular surfaces in guiding the joint motion. Proceedings of the Institution of Mechanical Engineers Part H, Journal of engineering in medicine. 2007;221(8):821–32. . 18161242 10.1243/09544119JEIM181

[85] Jeong WS, Yoo YS, Kim DY, Shetty NS, Smolinski P, Logishetty K, et al An analysis of the posterior cruciate ligament isometric position using an in vivo 3-dimensional computed tomography-based knee joint model. Arthroscopy: the journal of arthroscopic & related surgery: official publication of the Arthroscopy Association of North America and the International Arthroscopy Association. 2010;26(10):1333–9. 10.1016/j.arthro.2010.02.016 . 20887932

[86] Markolf KL, O'Neill G, Jackson SR, McAllister DR. Effects of applied quadriceps and hamstrings muscle loads on forces in the anterior and posterior cruciate ligaments. The American journal of sports medicine. 2004;32(5):1144–9. 10.1177/0363546503262198 . 15262635

[87] Markolf KL, Feeley BT, Tejwani SG, Martin DE, McAllister DR. Changes in knee laxity and ligament force after sectioning the posteromedial bundle of the posterior cruciate ligament. Arthroscopy: the journal of arthroscopic & related surgery: official publication of the Arthroscopy Association of North America and the International Arthroscopy Association. 2006;22(10):1100–6. 10.1016/j.arthro.2006.05.018 . 17027408

[88] Markolf KL, Feeley BT, Jackson SR, McAllister DR. Where should the femoral tunnel of a posterior cruciate ligament reconstruction be placed to best restore anteroposterior laxity and ligament forces? The American journal of sports medicine. 2006;34(4):604–11. 10.1177/0363546505281809 . 16365374

[89] Oakes DA, Markolf KL, McWilliams J, Young CR, McAllister DR. The effect of femoral tunnel position on graft forces during inlay posterior cruciate ligament reconstruction. The American journal of sports medicine. 2003;31(5):667–72. . 12975184 10.1177/03635465030310050601

[90] Wang CJ, Walker PS. The effects of flexion and rotation on the length patterns of the ligaments of the knee. Journal of biomechanics. 1973;6(6):587–96. . 4757478 10.1016/0021-9290(73)90016-x

[91] Wang CJ, Chen HH, Chen HS, Huang TW. Effects of knee position, graft tension, and mode of fixation in posterior cruciate ligament reconstruction: a cadaveric knee study. Arthroscopy: the journal of arthroscopic & related surgery: official publication of the Arthroscopy Association of North America and the International Arthroscopy Association. 2002;18(5):496–501. 10.1053/jars.2002.32326 . 11987060

[92] Wismans J, Veldpaus F, Janssen J, Huson A, Struben P. A three-dimensional mathematical model of the knee-joint. Journal of biomechanics. 1980;13(8):677–85. . 7419534 10.1016/0021-9290(80)90354-1

[93] Harner CD, Janaushek MA, Ma CB, Kanamori A, Vogrin TM, Woo SL. The effect of knee flexion angle and application of an anterior tibial load at the time of graft fixation on the biomechanics of a posterior cruciate ligament-reconstructed knee. The American journal of sports medicine. 2000;28(4):460–5. . 10921635 10.1177/03635465000280040401

[94] Harner CD, Vogrin TM, Hoher J, Ma CB, Woo SL. Biomechanical analysis of a posterior cruciate ligament reconstruction. Deficiency of the posterolateral structures as a cause of graft failure. The American journal of sports medicine. 2000;28(1):32–9. . 10653541 10.1177/03635465000280011801

[95] Margheritini F, Rihn JA, Mauro CS, Stabile KJ, Woo SL, Harner CD. Biomechanics of initial tibial fixation in posterior cruciate ligament reconstruction. Arthroscopy: the journal of arthroscopic & related surgery: official publication of the Arthroscopy Association of North America and the International Arthroscopy Association. 2005;21(10):1164–71. 10.1016/j.arthro.2005.06.017 . 16226642

[96] Markolf KL, Graves BR, Sigward SM, Jackson SR, McAllister DR. Effects of posterolateral reconstructions on external tibial rotation and forces in a posterior cruciate ligament graft. The Journal of bone and joint surgery American volume. 2007;89(11):2351–8. 10.2106/JBJS.F.01086 . 17974876

[97] Markolf KL, Slauterbeck JL, Armstrong KL, Shapiro MM, Finerman GA. Effects of combined knee loadings on posterior cruciate ligament force generation. Journal of orthopaedic research: official publication of the Orthopaedic Research Society. 1996;14(4):633–8. 10.1002/jor.1100140419 . 8764874

[98] Lenschow S, Zantop T, Weimann A, Lemburg T, Raschke M, Strobel M, et al Joint kinematics and in situ forces after single bundle PCL reconstruction: a graft placed at the center of the femoral attachment does not restore normal posterior laxity. Archives of orthopaedic and trauma surgery. 2006;126(4):253–9. 10.1007/s00402-005-0062-9 . 16273379

[99] Shelburne KB, Pandy MG. Determinants of cruciate-ligament loading during rehabilitation exercise. Clinical biomechanics. 1998;13(6):403–13. . 11415815 10.1016/s0268-0033(98)00094-1

[100] Belbasis A, Fuss FK, Sidhu J. Estimation of Cruciate Ligament Forces Via Smart Compression Garments. Procedia Engineering. 2015;112:169–74.

[101] Collins JJ, O'Connor. Muscle-ligament interactions at the knee during walking. Proceedings of the Institution of Mechanical Engineers Part H, Journal of engineering in medicine. 1991;205(1):11–8. . 1670070 10.1243/PIME_PROC_1991_205_256_02

[102] Harrington IJ. A bioengineering analysis of force actions at the knee in normal and pathological gait. Biomedical engineering. 1976;11(5):167–72. . 1276337

[103] Hu CC, Lu TW, Chen SC. Influence of model complexity and problem formulation on the forces in the knee calculated using optimization methods. Biomedical engineering online. 2013;12:20 10.1186/1475-925X-12-20 23496903 PMC3606467

[104] Shelburne KB, Pandy MG, Anderson FC, Torry MR. Pattern of anterior cruciate ligament force in normal walking. Journal of biomechanics. 2004;37(6):797–805. 10.1016/j.jbiomech.2003.10.010 . 15111067

[105] Harner CD, Hoher J, Vogrin TM, Carlin GJ, Woo SL. The effects of a popliteus muscle load on in situ forces in the posterior cruciate ligament and on knee kinematics. A human cadaveric study. The American journal of sports medicine. 1998;26(5):669–73. . 9784814 10.1177/03635465980260051201

[106] Li G, Most E, DeFrate LE, Suggs JF, Gill TJ, Rubash HE. Effect of the posterior cruciate ligament on posterior stability of the knee in high flexion. Journal of biomechanics. 2004;37(5):779–83. 10.1016/j.jbiomech.2003.09.031 . 15047008

[107] Nagura T, Dyrby CO, Alexander EJ, Andriacchi TP. Mechanical loads at the knee joint during deep flexion. J Orthopaed Res. 2002;20(4):881–6. 10.1016/S0736-0266(01)00178-4 12168682

[108] Alkjaer T, Wieland MR, Andersen MS, Simonsen EB, Rasmussen J. Computational modeling of a forward lunge: towards a better understanding of the function of the cruciate ligaments. J Anat. 2012;221(6):590–7. 23057673 10.1111/j.1469-7580.2012.01569.x PMC3512282

[109] Moro-oka TA, Hamai S, Miura H, Shimoto T, Higaki H, Fregly BJ, et al Dynamic activity dependence of in vivo normal knee kinematics. Journal of orthopaedic research: official publication of the Orthopaedic Research Society. 2008;26(4):428–34. 10.1002/jor.20488 . 17985389

[110] Hefzy MS, al Khazim M, Harrison L. Co-activation of the hamstrings and quadriceps during the lunge exercise. Biomedical sciences instrumentation. 1997;33:360–5. . 9731386

[111] Hefzy MS, Grood ES. Sensitivity of insertion locations on length patterns of anterior cruciate ligament fibers. Journal of biomechanical engineering. 1986;108(1):73–82. . 3959555 10.1115/1.3138583

[112] Gupte CM, Bull AM, Thomas RD, Amis AA. The meniscofemoral ligaments: secondary restraints to the posterior drawer. Analysis of anteroposterior and rotary laxity in the intact and posterior-cruciate-deficient knee. J Bone Joint Surg Br. 2003;85(5):765–73. . 12892207

[113] Speirs AD, Heller MO, Duda GN, Taylor WR. Physiologically based boundary conditions in finite element modelling. Journal of biomechanics. 2007;40(10):2318–23. 10.1016/j.jbiomech.2006.10.038 . 17166504

[114] Wu G, Siegler S, Allard P, Kirtley C, Leardini A, Rosenbaum D, et al ISB recommendation on definitions of joint coordinate system of various joints for the reporting of human joint motion—part I: ankle, hip, and spine. International Society of Biomechanics. J Biomech. 2002;35(4):543–8. . 11934426 10.1016/s0021-9290(01)00222-6

