# Supplementary material for: Loading Patterns of the Posterior Cruciate Ligament in the Healthy Knee: A Systematic Review
Source: PLoS One. 2016 Nov 23;11(11):e0167106. doi: 10.1371/journal.pone.0167106 (PMC5120848; doi:10.1371/journal.pone.0167106)
Supplement: S1 File — (DOC) [file pone.0167106.s001.doc]

**Search string in PubMed:**

Search **(((((((((((((((cruciate ligament[Title/Abstract]) OR collateral ligament[Title/Abstract])) AND load[Title/Abstract])) OR ((((cruciate ligament[Title/Abstract]) OR collateral ligament[Title/Abstract])) AND force[Title/Abstract])) OR ((((cruciate ligament[Title/Abstract]) OR collateral ligament[Title/Abstract])) AND tension[Title/Abstract])) OR ((((cruciate ligament[Title/Abstract]) OR collateral ligament[Title/Abstract])) AND strain[Title/Abstract])) OR ((((cruciate ligament[Title/Abstract]) OR collateral ligament[Title/Abstract])) AND stress[Title/Abstract])) OR ((((cruciate ligament[Title/Abstract]) OR collateral ligament[Title/Abstract])) AND length[Title/Abstract])) OR ((((cruciate ligament[Title/Abstract]) OR collateral ligament[Title/Abstract])) AND lengthening[Title/Abstract])) OR ((((cruciate ligament[Title/Abstract]) OR collateral ligament[Title/Abstract])) AND elongation[Title/Abstract])) OR ((((cruciate ligament[Title/Abstract]) OR collateral ligament[Title/Abstract])) AND isometry[Title/Abstract])) AND full text[sb] AND Humans[Mesh] AND English[lang])) OR (((((((((((((knee[Title/Abstract]) AND ligament[Title/Abstract]) AND isometry[Title/Abstract])) OR (((knee[Title/Abstract]) AND ligament[Title/Abstract]) AND elongation[Title/Abstract])) OR (((knee[Title/Abstract]) AND ligament[Title/Abstract]) AND lengthening[Title/Abstract])) OR (((knee[Title/Abstract]) AND ligament[Title/Abstract]) AND length[Title/Abstract])) OR (((knee[Title/Abstract]) AND ligament[Title/Abstract]) AND stress[Title/Abstract])) OR (((knee[Title/Abstract]) AND ligament[Title/Abstract]) AND strain[Title/Abstract])) OR (((knee[Title/Abstract]) AND ligament[Title/Abstract]) AND tension[Title/Abstract])) OR (((knee[Title/Abstract]) AND ligament[Title/Abstract]) AND force[Title/Abstract])) OR (((knee[Title/Abstract]) AND ligament[Title/Abstract]) AND load[Title/Abstract])) AND full text[sb] AND Humans[Mesh] AND English[lang])** Filters: **Full text; Humans; English**
